# Comorbidity burden in elderly high-grade glioma patients: impact on radiotherapy outcomes

**DOI:** 10.1186/s12885-025-14957-5

**Published:** 2025-10-01

**Authors:** Sophia M. Leiss, Benedikt Wiestler, Helen X. Hou, Daniel Schmottermeyer, Valeriya Sackerer, Christian Diehl, Jan Peeken, Kai Borm, Chiara Negwer, Arthur Wagner, Igor Yakushev, Claire Delbridge, Meike Mitsdörffer, Friederike Schmidt-Graf, Bernhard Meyer, Stephanie Combs, Denise Bernhardt

**Affiliations:** 1 Department of Radiation Oncology, TUM School of Medicine and Health, TUM University Hospital Rechts der Isar, Technical University of Munich, Munich, Germany; 2 Department of Neuroradiology, TUM School of Medicine and Health, TUM University Hospital Rechts der Isar, Technical University of Munich, Munich, Germany; 3 TranslaTUM, TU Munich, 81675 Munich, Germany; 4 Department of Neurosurgery, TUM School of Medicine and Health, TUM University Hospital Rechts der Isar, Technical University of Munich, Munich, Germany; 5 Department of Nuclear Medicine, TUM School of Medicine and Health, TUM University Hospital Rechts der Isar, Technical University of Munich, Munich, Germany; 6 Department of Pathology and Neuropathology, TUM School of Medicine, TU Munich, 81675 Munich, Germany; 7 Department of Neurology, TUM School of Medicine and Health, TUM University Hospital Rechts der Isar, Technical University of Munich, Munich, Germany; 8 Institute of Radiation Medicine (IRM), Helmholtz Zentrum München (HMGU), Munich, Germany; 9 German Consortium for Translational Cancer Research (DKTK), Partner Site Munich, Munich, Germany

**Keywords:** Comorbidities, Glioblastoma, Radiotherapy, Chemotherapy, Survival outcomes

## Abstract

**Background:**

Elderly high-grade glioma (HGG) or glioblastoma (GBM) patients face challenging treatment conditions due to comorbidities and age-related factors. The age-adjusted Charlson Comorbidity Index (ACCI) accounts for age and comorbidities and serves as a tool for predicting survival rates in various clinical scenarios. This study examined its prognostic value in elderly HGG patients undergoing radiotherapy (RT) and concurrent chemoradiotherapy (CRT).

**Methods:**

We retrospectively analyzed 163 elderly HGG patients (≥ 60 years) treated with radiotherapy (RT) or chemo-RT (CRT) at TUM University Hospital (2001–2021). Kaplan-Meier analysis estimated median overall survival (OS) by ACCI group (≤ 5 vs. ≥6). Multivariate Cox regressions assessed OS and progression-free-survival (PFS) based on fractionation and treatment strategies. Further Cox models evaluated ACCI scores, age, comorbidities, and mortality. A random survival forest (RSF) identified key survival predictors, using permutation importance with bootstrapped confidence intervals.

**Results:**

Among the 163 HGG patients, those with greater comorbidities (ACCI ≥ 6) had a shorter median OS (14.8 months) than did those with ACCI ≤ 5 (22.6 months) (log-rank *p* = 0.463). In the ACCI ≤ 5 subgroup, hypofractionated RT (hRT) alone was significantly associated with worse OS than Stupp was (HR = 85.7, 95% CI: 7.1-914.3, *p* = 0.0004), whereas no significant differences were detected in the ACCI ≥ 6 subgroup. Hypofractionated RT was associated with improved PFS in patients with an ACCI ≥ 6 (HR = 0.47, 95% CI: 0.24–0.92, *p* = 0.027), and MGMT methylation better predicted OS (HR = 0.31, *p* = 0.0039) and PFS (HR = 0.32, *p* = 0.0059). Diabetes without complications independently predicted worse OS (HR = 2.91 (95% CI: 1.63–5.18, *p* < 0.001)) and PFS (HR = 2.59 (95% CI: 1.43–4.70, *p* = 0.002), with a significant interaction between diabetes and the ACCI (HR = 0.26, 95% CI: 0.07–0.91, *p* = 0.03). RSF models identified age as the key predictor of OS and MGMT methylation as the main predictor of PFS, while ACCI ≥ 6 contributed only modestly (mean drop for OS: 0.025; and PFS: 0.019).

**Conclusions:**

The ACCI showed limited and inconsistent prognostic value in elderly glioblastoma patients, while diabetes emerged as the only consistent comorbidity predictor of OS and PFS. These findings suggest that comorbidity burden may influence outcomes but underscore the need for larger studies to clarify the role of the ACCI in treatment stratification.

**Supplementary Information:**

The online version contains supplementary material available at 10.1186/s12885-025-14957-5.

## Background

Glioblastoma (GBM) is the most common (49%) and aggressive primary brain tumor in adults. Maximal safe resection followed by concurrent chemoradiotherapy (CRT) and adjuvant temozolomide (the Stupp protocol) remains the standard of care [1], significantly improving 2-year survival by 26.5% [1]. However, the prognosis remains poor, with a 5-year survival rate of only 4.1%, and recurrence is almost inevitable despite aggressive first-line treatment [2]. Choosing the optimal treatment approach - hypofractionated RT (hRT), normofractionated RT (normoRT), or TMZ alone—is challenging and must balance efficacy, tolerability, and patient quality of life. Treatment selection in elderly glioblastoma patients remains complex and should be guided by functional status and molecular markers rather than chronological age, as emphasized in the EANO guidelines on diffuse gliomas [3]. This is further supported by evidence from the CE.6 trial, which demonstrated improved survival with the addition of temozolomide to short-course radiotherapy in elderly patients [4].

In addition to treatment challenges, comorbidities frequently complicate GBM management, particularly in elderly patients [5]. A population-based study reported that GBM patients have a markedly increased risk of seizures, venous thromboembolism, and cardiovascular disease, highlighting the burden of comorbidities in this population [6]. Additionally, asthma and hypercholesterolemia have been linked to shorter survival [7], reinforcing the need for comorbidity assessment in treatment decisions.

The Age-Adjusted Charlson Comorbidity Index (ACCI) is a widely used risk stratification tool in oncology that integrates both age and comorbidities to assess prognosis [8–13]. While extensively validated in other malignancies, its prognostic value in elderly GBM patients undergoing radiotherapy or chemoradiotherapy remains unclear.

This study aims to evaluate the ACCI in this patient group to refine risk assessment and improve clinical decision-making.

## Methods

### Patient cohort and treatment

We conducted a retrospective analysis of 163 elderly patients diagnosed with glioblastoma between 2001 and 2021 at TUM University Hospital. As diagnoses were made according to historical WHO classifications, the cohort includes both IDH-wildtype and IDH-mutant tumors, all of which were classified as WHO grade 4 at the time of diagnosis. All patients were aged ≥ 61 years at initial diagnosis and received either radiotherapy (RT) alone or concurrent chemoradiotherapy (CRT) per standard protocols. The choice between hypofractionated and normofractionated RT was based on clinical judgment, primarily considering patient age, performance status, and comorbidities, in line with EANO guidelines. Frail or older patients more frequently received hypofractionated regimens.

In this cohort, the average age at first diagnosis was 70.38 years (SD = 6.83), with 77 female patients. The mean Karnofsky performance status (KPS) at the initial evaluation was 70, and the median ACCI was 5.0. A total of 116 patients (71.2%) died at the time of analysis. Molecular profiling revealed that 52 patients had MGMT promoter methylation. Ten patients had an IDH1 mutation but were diagnosed as glioblastoma (WHO grade 4) according to the historical classification in use at the time. Surgical intervention for tumor recurrence was performed in 48 patients, and 33 patients underwent reirradiation (Re-RT). Additionally, 5 patients received tumor-treating fields (TTFs). Chemotherapy beyond first-line treatment was recorded in 53 patients for second-line therapy, 13 for third-line therapy, and 4 for fourth-line chemotherapy. For this study, “multiple lines of chemotherapy” refers to any patient who received two or more distinct systemic chemotherapy regimens beyond the initial concurrent or adjuvant temozolomide course, regardless of drug class or combination.

With respect to treatment-related complications, 31 patients developed motor disorders, 13 had sensory disorders, 14 experienced epilepsy, 10 developed leukopenia, 16 had thrombocytopenia, and 117 experienced other toxicities within six months of the first radiotherapy initiation. See Table 1 for details.

### Comorbidity assessment

The ACCI is a widely used tool for risk stratification in various diseases [14–20] and was adapted from the Charlson Comorbidity Index (CCI) [21]. It assigns weighted scores to specific comorbid conditions, such as myocardial infarction, congestive heart failure, peripheral vascular disease, dementia, liver disease, and diabetes, while also incorporating an adjustment for patient age. This scoring system enhances prognostic accuracy by accounting for both disease burden and age-related risk.

The ACCI was calculated for all patients on the basis of previously established weighting criteria (detailed in Supplementary Table 1). Given the absence of a universally accepted ACCI cutoff for HGG, we categorized patients into ACCI ≤ 5 and ACCI ≥ 6 groups to ensure a balanced distribution for statistical comparison. A lower threshold would have resulted in highly unequal groups, reducing statistical power and comparability. This data-driven stratification aimed to optimize group sizes while maintaining clinical relevance in assessing the impact of comorbidities on survival outcomes.

Like Barz et al. (2022) [22], to evaluate the validity of this cutoff, we assessed its ability to predict survival via receiver operating characteristic curve analysis at 6-month and 1-year survival time points. The area under the curve was 0.60 for 1-year survival and 0.61 for 6-month survival, indicating limited discriminative ability. The sensitivity and specificity at 6 months were 0.68 and 0.53, respectively, whereas at 1 year, they were 0.63 and 0.57, respectively. These findings suggest that while the ACCI cutoff provides some stratification, comorbidity alone is not a sufficient predictor of survival in glioblastoma patients, highlighting the need for multivariable risk models, as outlined in the following analysis.

### Statistical analysis

All analyses were conducted in Python 3.13.1 via Jupyter Notebook. Data preprocessing was performed with pandas (v2.2.3) and numpy (v2.2.1). Survival analyses, including Kaplan‒Meier estimates and Cox proportional hazards models, were performed using lifelines (v0.30.0), with log-rank and Kruskal‒Wallis tests via scipy (v1.15.1).

Kaplan‒Meier analysis was used to estimate the median OS and PFS across ACCI groups, with differences tested via the log-rank test. Multivariate Cox regression models were used to assess the impact of the ACCI, KPS, MGMT methylation, and treatment strategies (e.g., hRT vs. normoRT, CRT combinations) on OS and PFS. Model performance was evaluated via Harrell’s C-index. For sensitivity analyses, we additionally adjusted Cox models for KPS, EoR, and MGMT methylation; these adjustments did not materially alter the main results. IDH mutation status was not included in Cox models, as it was rare (*n* = 10) and mostly missing in this elderly HGG cohort, consistent with the known biology of these tumors. We employed a random survival forest (RSF) model (*sksurv.ensemble. RandomSurvivalForest*) to identify key predictors of OS and PFS. Feature importance was assessed via permutation importance (*sklearn.inspection.permutation_importance*), with bootstrapped confidence intervals (*sklearn.utils.resample*). The model included variables related to tumor biology (MGMT methylation), treatment factors (recurrence resection, reirradiation, and multiple chemotherapy lines), and patient characteristics (age at first diagnosis and comorbidity burden represented by ACCI binary categorization). The results were visualized via *matplotlib* and *seaborn*.

All patients underwent regular clinical and imaging follow-ups during and after RT or CRT. Hypofractionation included fractions between 5 and 15, whereas normoRT included all patients who received a single dose of either 1,8 Gy–2 Gy. The category “Others” was created for patients who had a change in the RT schema, e.g., due to worsening of symptoms. The first posttreatment assessment occurred 4–6 weeks after therapy completion, followed by routine MRI and neurological evaluations every 2–3 months. Each visit included a comprehensive neurological examination, MRI, and a review of clinical symptoms to monitor tumor progression and guide further treatment decisions.

**Table 1:** Patient and treatment characteristics

	Overall
M: F ratio (%)	86:77 (52.8%: 47.2%)
Age at first diagnosis mean *(*± SD, range)	70.38 (± 6.83, 61.10-85.54)
Dead (%)	116/163 (71.2%)
ACCI median (range)	5.0/163 (5.0–13.0)
Genetics	
MGMT methylated	52/158 (32.9%)
IDH1 mutated	10/156 (6.4%)
KPS at diagnosis	
Mean KPS	70
KPS < 50	7/141 (5.0%)
KPS 50–60	13/141 (9.2%)
KPS 70–80	65/141 (46.1%)
KPS > 80	52/141 (36.9%)
1 st RT	
Fraction categories	
Hypofractionated (5–15 fractions)	51/163 (31.3%)
Normofractionated	100/163 (61.3%)
1 st C(RT)	
hRT alone	35/118 (29.7%)
concurrent hRT with TMZ	16/118 (13.6%)
normofractionated RT (60 Gy) + TMZ (Stupp)	67/118 (56.8%)
Impairments < 6 months post-RT	
Motor disorders (POST-RT)	31/163 (19.0%)
Sensory disorders (POST-RT)	13/163 (8.0%)
Epilepsy (POST-RT)	14/163 (8.6%)
Leukopenia (POST-RT)	10/163 (6.1%)
Thrombocytopenia (POST-RT)	16/163 (9.8%)
Other toxicity (POST-RT)	117/163 (71.8%)
Recurrence treatment	
Recurrence Resection	48/160 (30.0%)
Re-RT	33/159 (20.8%)
2.Line ChT	53/163 (32.5%)
3.Line ChT	13/163 (8.0%)
4.Line ChT	4/163 (2.5%)
Bevacizumab	9/163 (5.5%)
TTF	5/163 (3.2%)

## Results

**Fig. 1:**
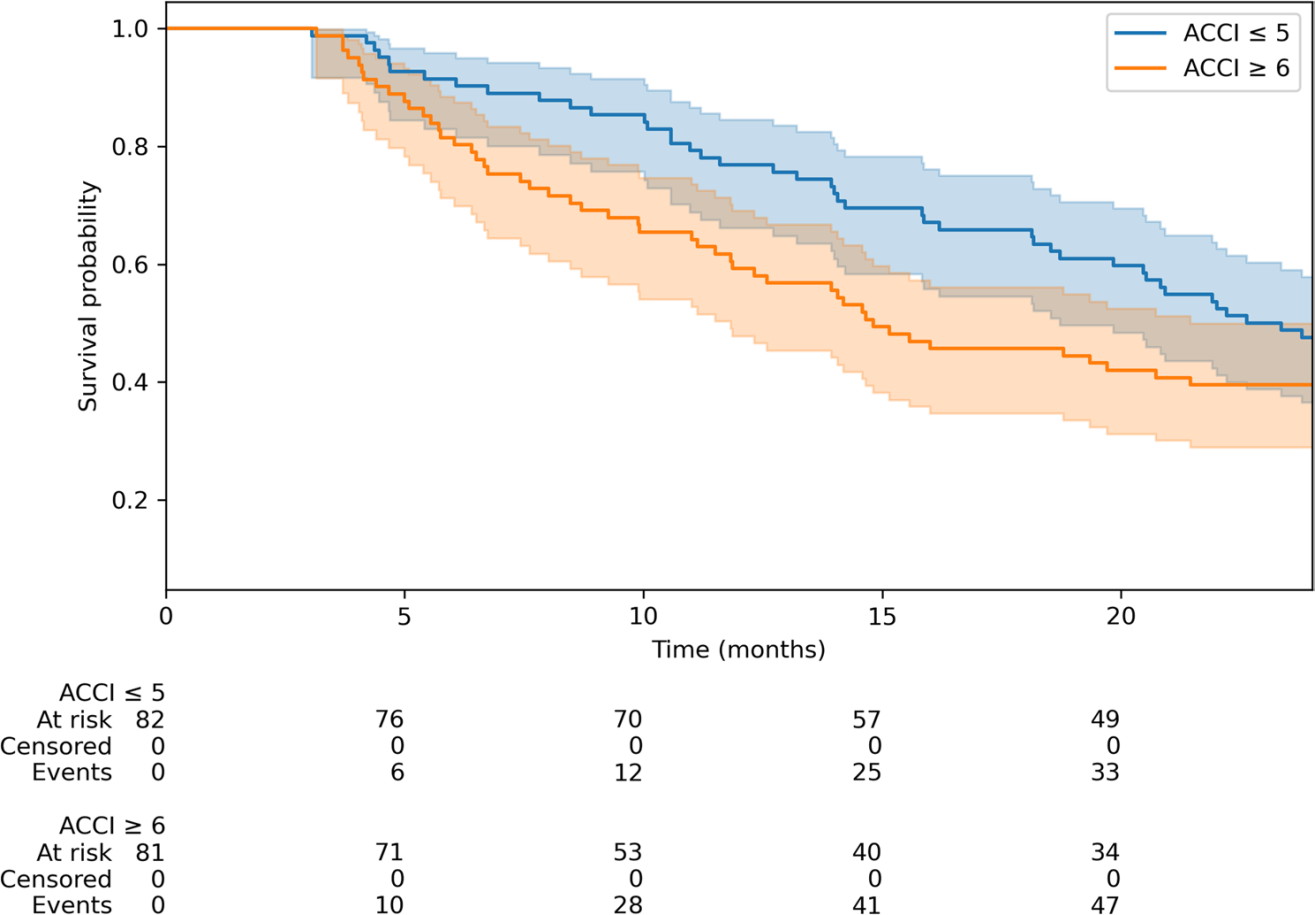
OS in patients with an age-adjusted ACCI ≤ 5, compared with patients with an ACCI ≤ 6 (Kaplan‒Meier estimation) (median 22.6 vs. 14.8 months; log-rank test *p* = 0.4631)

Among the 163 patients included in the study, 82 had an ACCI score of ≤ 5, whereas 81 had an ACCI score of ≥ 6. K‒M analysis revealed a median OS of 22.6 months for the ACCI ≤ 5 group, whereas the median OS of 14.8 months for the ACCI ≥ 6 group was not significantly different (*p* = 0.46) (Fig. 1).

### ACCI and treatment combinations

Cox regression models were conducted separately for patients with low (ACCI ≤ 5) and high (ACCI ≥ 6) comorbidity scores, using *normofractionated RT (60 Gy) + TMZ (Stupp)* as the reference category. In the ACCI ≤ 5 group, patients treated with hRT alone had significantly worse OS than those treated with the Stupp regimen did (HR = 85.7, 95% CI: 7.1-914.3, *p* = 0.0004). No statistically significant difference in OS was observed for patients receiving concurrent hRT with TMZ (HR = 0.71, 95% CI: 0.25-2.00, *p* = 0.51), although a trend toward improved survival was noted. For PFS, neither hRT combined with TMZ (HR = 0.83, 95% CI: 0.29–2.33, *p* = 0.72) nor hRT alone (HR = 1.22, 95% CI: 0.61–2.99, *p* = 0.67) differed significantly, but there was a trend toward poorer PFS in the hRT alone group.

**Fig. 2:**
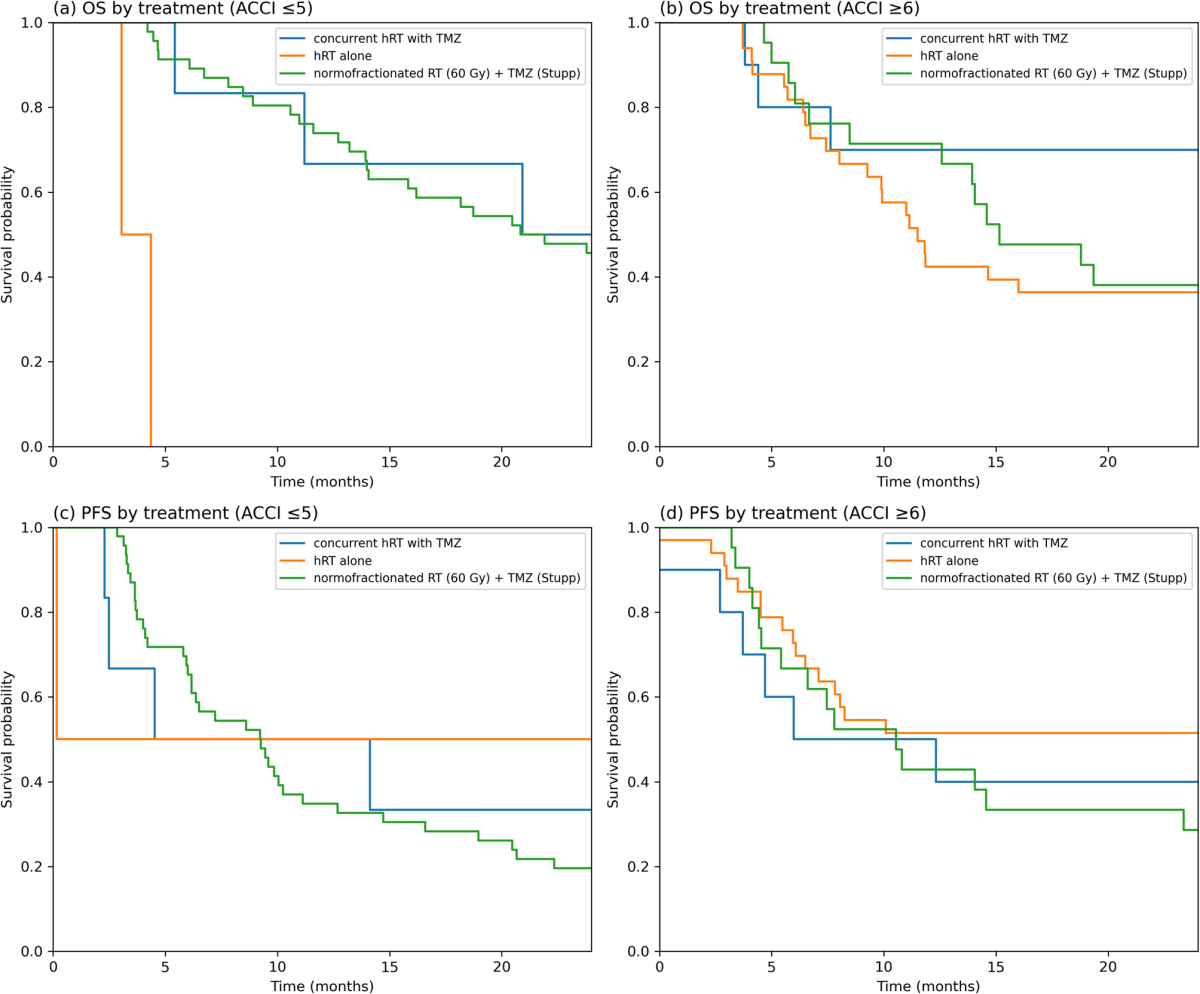
Kaplan Meier curves for OS and PFS by treatment and comorbidity group

In the ACCI ≥ 6 group, although not statistically significant, patients receiving concurrent hRT with TMZ showed a trend toward a better OS than did those receiving Stupp (HR = 0.51, 95% CI: 0.17–1.53, *p* = 0.23). Patients treated with hRT alone were not significantly different (HR = 1.16, 95% CI: 0.28–4.48, *p* = 0.65). Similarly, for PFS, hRT combined with TMZ (HR = 0.94, 95% CI: 0.37–2.44, *p* = 0.90) and hRT alone (HR = 0.65, 95% CI: 0.32–1.32, *p* = 0.23) did not significantly differ from Stupp alone, but the direction of effect suggested that hRT alone may benefit PFS in this high-comorbidity group (Fig.2). Sensitivity analyses additionally adjusting for Karnofsky Performance Status, MGMT promoter methylation, and extent of resection yielded similar results, with no significant differences between treatment strategies within ACCI groups.

### ACCI and hRT vs. normoRT

**Fig. 3:**
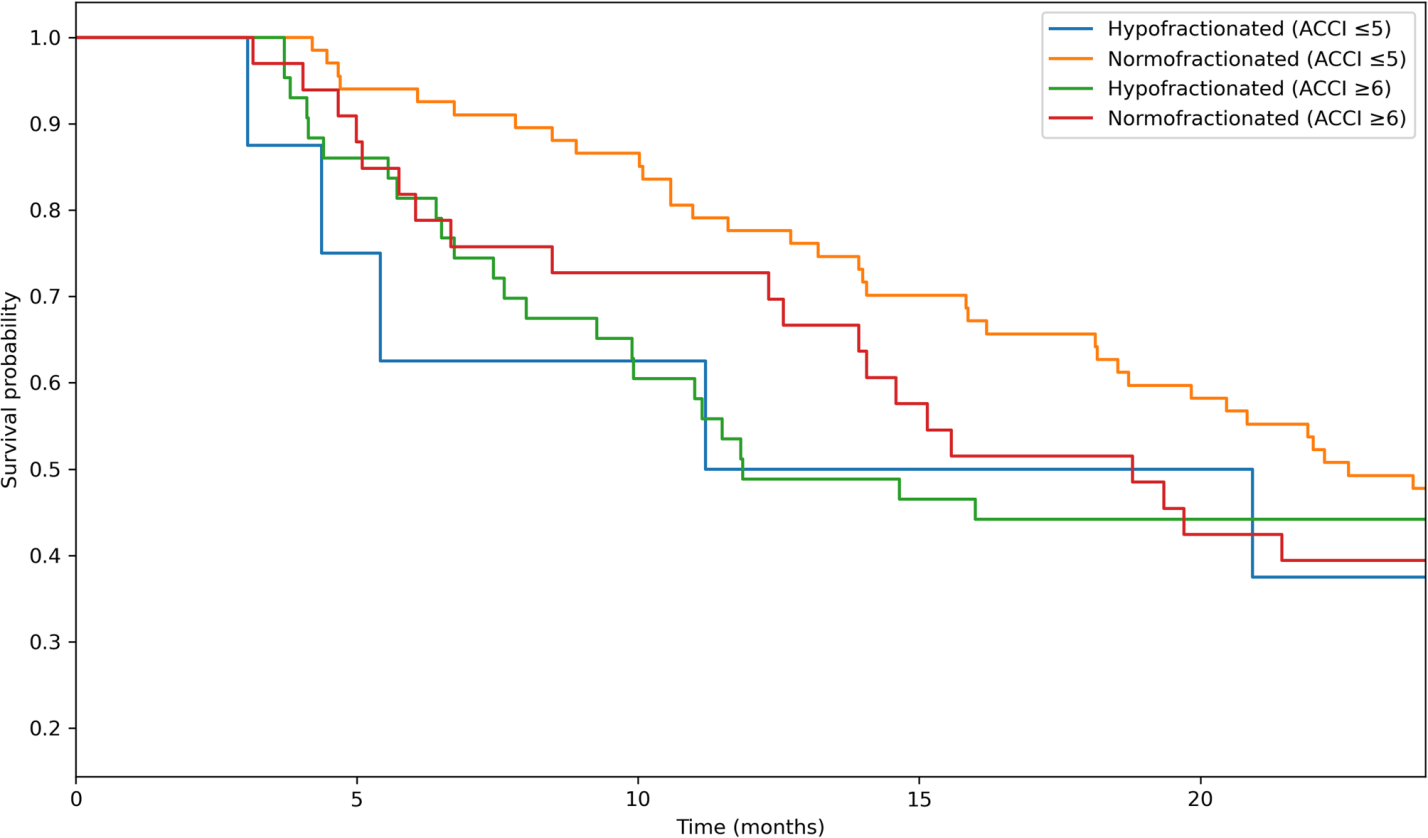
Overall survival by radiotherapy fractionation and ACCI group

In the comparison between hypofractionated and normofractionated RT, no significant differences in OS were observed in either comorbidity subgroup. Among patients with an ACCI ≤ 5, the Cox regression yielded a HR of 1.27 (95% CI: 0.54–2.97, *p* = 0.583). and the log-rank test confirmed a nonsignificant difference (*p* = 0.5815). Similarly, in the ACCI ≥ 6 group, the HR was 0.96 (95% CI: 0.56–1.66, *p* = 0.884), with a log-rank p value of 0.8828. These findings suggest that the fractionation scheme alone does not significantly impact OS across comorbidity strata (Fig. 3). In the ACCI ≤5 group, no significant difference in PFS was observed between the hypofractionated and normofractionated radiotherapy groups (HR: 0.86, 95% CI: 0.34-2.15, p = 0.745). Among patients with greater comorbidities (ACCI ≥6), hypofractionation was associated with a nonsignificant trend toward improved PFS (HR: 0.68, 95% CI: 0.38-1.21, p = 0.192). Sensitivity analyses additionally adjusting for Karnofsky Performance Status, MGMT promoter methylation, and extent of resection yielded similar results, with no significant differences in OS or PFS between hypofractionated and normofractionated RT across comorbidity groups.

### Cox proportional hazards models of comorbidity and survival

**Fig. 4:**
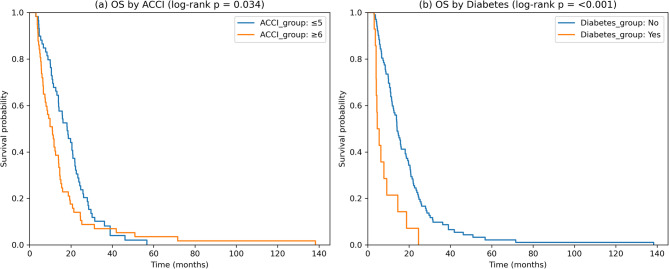
Kaplan-Meier curves for ACCI and diabetes in relation to OS**a** OS by ACCI (≤ 5 vs. ≥6). **b** OS by diabetes without complications

Kaplan-Meier analyses confirmed that ACCI was not significantly associated with OS or PFS whereas diabetes was associated with significantly worse OS and PFS (Fig. 4a and b). To further examine these associations while avoiding redundancy between the age-adjusted Charlson comorbidity index (ACCI) and its components, we prespecified two complementary Cox models.

### Model A (ACCI only)

When ACCI was modeled as a continuous variable without age or its components, it was not significantly associated with OS (HR 1.08, 95% CI 0.95–1.23, *p* = 0.22; concordance 0.589).

### Model B (components: age + diabetes, no ACCI)

In contrast, when age and diabetes were modeled separately, diabetes without complications was significantly associated with worse OS (HR 2.91, 95% CI 1.63–5.18, *p* < 0.001), while age was not statistically significant (HR 1.02 per year, 95% CI 0.99–1.06, *p* = 0.15). Model B showed higher discriminative ability (concordance 0.622) compared to Model A.

### Sensitivity analyses

In a model including the Charlson index without age plus age, neither variable was significant (concordance 0.579). In a model including ACCI with the diabetes weight removed plus diabetes, diabetes remained strongly associated with OS (HR 3.08, 95% CI 1.74–5.47, *p* < 0.001), while the adjusted ACCI component was not significant (concordance 0.612). These findings confirm that the prognostic effect was driven primarily by diabetes rather than the composite score.

Looking at PFS, in the ACCI-only model (Model A), the ACCI was not significantly associated with PFS (HR 1.12, 95% CI 0.95–1.32, *p* = 0.17; concordance 0.544). In the component model (Model B), diabetes without complications was significantly associated with shorter PFS (HR 2.59, 95% CI 1.43–4.70, *p* = 0.002), while age was not significant (HR 1.02, 95% CI 0.99–1.05, *p* = 0.30; concordance 0.565).

Sensitivity analyses confirmed these findings. A model including CCI without age plus age showed no significant associations (concordance 0.538). A model including ACCI with the diabetes weight removed plus diabetes confirmed the adverse prognostic impact of diabetes (HR 2.64, 95% CI 1.45–4.81, *p* = 0.002), whereas the modified ACCI remained non-significant (concordance 0.558).

In the exploratory RSF analysis for OS (C-index: 0.697), age at first diagnosis was the strongest predictor (mean drop: 0.1160, SD: 0.0214), followed by diabetes without complications (0.0516, SD: 0.0156). The ACCI total score showed limited importance (0.0201, SD: 0.0138), and the binary ACCI group had negligible impact (0.0070, SD: 0.0043). When age was excluded (C-index: 0.611), the ACCI total score (0.0316, SD: 0.0213) and diabetes (0.0243, SD: 0.0128) emerged as the main predictors, with the binary ACCI again negligible.

For PFS, RSF performance was weaker (C-index: 0.663 with age; 0.539 without age). With age included, age was again the dominant predictor (0.1241, SD: 0.0434), followed by diabetes (0.0300, SD: 0.0115), while the ACCI total score had minimal contribution (0.0065, SD: 0.0079). Without age, predictive accuracy dropped markedly, with diabetes (0.0374, SD: 0.0191) carrying the only relevant signal and ACCI measures showing little value.

### Effects of kaplan‒meier survival with post-RT impairment on OS and PFS

Post-RT impairments were relatively common, with motor disorders (19%), epilepsy (8.6%), and thrombocytopenia (9.8%) being the most frequently observed (Table 1). These variables were included in survival analyses but did not show significant associations with OS or PFS: epilepsy HR 1.44 (95% CI 0.74–2.81, *p* = 0.28), leukopenia HR 0.69 (0.25–1.88, *p* = 0.47), thrombocytopenia HR 0.93 (0.39–2.20, *p* = 0.87). For PFS, none reached significance; the largest effects were motor disorders HR 2.01 (0.97–4.16, *p* = 0.061) and epilepsy HR 1.50 (0.78–2.89, *p* = 0.23). Across treatment strategies—hRT alone, concurrent hRT + TMZ, and normofractionated RT (60 Gy) + TMZ (Stupp)—toxicity rates were similar within both ACCI ≤ 5 and ACCI ≥ 6 (all Fisher’s exact *p* ≥ 0.136); in ACCI ≥ 6, thrombocytopenia and “other toxicity” were numerically higher with Stupp but not significant.

### Cox model with interaction terms and RSF with genetic-related factors and recurrence treatments on OS

The model showed good discrimination (Concordance = 0.70), although it lacked statistical power. MGMT methylation (HR = 0.89, 95% CI: 0.68–1.18, *p* = 0.42), recurrence resection (HR = 0.76, 95% CI: 0.49–1.17, *p* = 0.21), Re-RT (HR = 0.71, 95% CI: 0.37–1.36, *p* = 0.30), and multiple chemotherapy lines (HR = 0.80, 95% CI: 0.44–1.46, *p* = 0.47) showed numerically protective trends. An ACCI ≥ 6 was associated with a nonsignificant trend toward worse survival (HR = 1.27, 95% CI: 0.78–2.07, *p* = 0.33). Interaction terms (ACCI × Re-RT, ACCI × chemo lines) were also nonsignificant (HR = 1.24, 95% CI: 0.43–3.52, *p* = 0.69; HR = 0.87, 95% CI: 0.30–2.47, *p* = 0.79, respectively).

We included age in the RSF model, as it handles multicollinearity robustly and can capture nonlinear effects, whereas in the Cox model, we excluded age to avoid collinearity with the ACCI score, which already incorporates age.

**Fig. 5:**
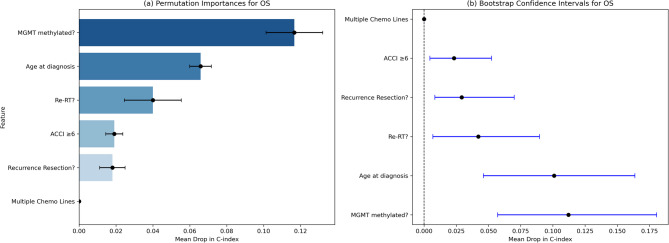
Feature Importance in Predicting OS. **a** Permutation importance, showing the mean decrease in the concordance index (C-index) when each variable is removed from the random survival forest model. **a **bootstrapped confidence intervals for permutation importance. Higher mean drops indicate stronger predictive value for survival

The RSF model achieved a concordance index of 0.735, indicating good discriminative performance. Age at first diagnosis was the strongest predictor of OS (mean decrease in the c-index = 0.057), followed by recurrence resection (0.048), Re-RT (0.041), MGMT promoter methylation (0.039), and ACCI ≥ 6 (0.026). Notably, receiving multiple lines of chemotherapy had no measurable contribution to the prediction in this model (mean drop = 0.000) (Fig. 5, a). Bootstrap confidence intervals (Fig. 5, b) confirmed that age at diagnosis (mean decrease: 0.057, 95% CI: 0.015–0.12), recurrence resection (0.052, 95% CI: 0.016–0.13), and Re-RT (0.041, 95% CI: 0.020–0.093) were the strongest predictors. MGMT methylation and an ACCI ≥ 6 had moderate contributions, whereas multiple chemotherapy lines had no predictive value.

### Cox model with interaction terms and RSF with genetic-related factors and recurrence treatments on PFS

In the Cox regression model for PFS, no variable reached statistical significance (concordance index 0.62). MGMT promoter methylation (HR = 0.81, 95% CI: 0.60–1.11, *p* = 0.19), Re-RT (HR = 0.67, 95% CI: 0.36–1.24, *p* = 0.20), and receiving multiple lines of chemotherapy (HR = 0.77, 95% CI: 0.43–1.38, *p* = 0.38) were not significantly associated with longer PFS. ACCI ≥ 6 (HR = 0.97, 95% CI: 0.58–1.63, *p* = 0.91) and recurrence resection (HR = 1.09, 95% CI: 0.71–1.66, *p* = 0.69) were also not significantly associated with outcomes. The interaction terms (ACCI × Re-RT and ACCI × Chemo) did not meaningfully alter the results.

**Fig. 6:**
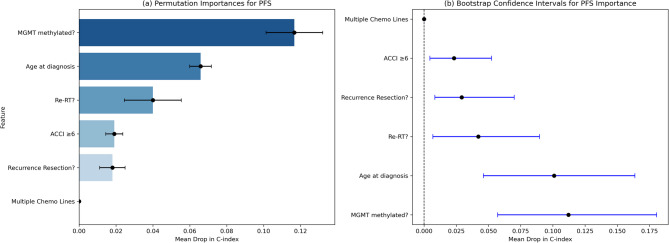
Feature Importance in Predicting PFS. **a** Permutation importance, showing the mean decrease in the concordance index (C-index) when each variable is removed from the random survival forest model. **b** bootstrapped confidence intervals for permutation importance. Higher mean drops indicate stronger predictive value for survival

The RSF model for predicting PFS achieved a concordance index of 0.72. MGMT promoter methylation was the strongest predictor (mean decrease: 0.117), followed by age at diagnosis (mean decrease: 0.057) and Re-RT (mean decrease: 0.040). An ACCI ≥ 6 contributed modestly (mean drop: 0.019), and recurrence resection had a limited influence (mean drop: 0.018), whereas multiple chemotherapy lines had no measurable effect (Fig. 6, a).

Bootstrapping supports the RSF results, confirming the stability and reliability of the identified key predictors for PFS. (Fig. 6, b).

The OS and PFS outcomes are compared in Tables 2 and 3.

**Table 2:** Comparing RSF permutation importance for OS and PFS to highlight ranked predictors. The mean drop describes the drop in the concordance interval

Variable	OS (Mean Drop)	OS (SD)	PFS (Mean Drop)	PFS (SD)
Recurrence Resection?	0.048	0.015	0.018	0.007
Re-RT?	0.041	0.009	0.040	0.015
Age at First Diagnosis	0.057	0,017	0.066	0.006
MGMT Methylated?	0.040	0.004	0.117	0.015
ACCI ≥ 6	0.025	0.010	0.019	0.005
Multiple Chemo	0.000	0.000	0.000	0.000

**Table 3:** RSF bootstrapping was compared for OS and PFS to highlight the statistical reliability of the predictors

Variable	OS (Mean Drop)	OS (95% CI)	PFS (Mean Drop)	PFS (95% CI)
Recurrence Resection?	0.0515	0.0137–0.1220	0,0.292	0.0084-0.0700
Re-RT?	0.0535	0.0182–0.1056	0.0422	0.0068–0.0896
Age at First Diagnosis	0.0939	0.048–0.1370	0.1010	0.0461–0.1637
MGMT Methylated?	0.0577	0.0170–0.1113	0.1121	0.0571–0.1805
ACCI ≥ 6	0.0329	0.0017–0.0908	0.0233	0.0045–0.0524
Multiple Chemo	0.0000	0.0000–0.0000	0.0000	0.000–0.000

## Discussion

Our findings suggest a complex relationship between the comorbidity burden, as measured by the ACCI score, and survival in elderly HGG patients. While the median OS was numerically shorter in patients with an ACCI ≥ 6 than in those with an ACCI ≤ 5 (14.8 vs. 22.6 months), this difference was not statistically significant. Furthermore, the ACCI was not identified as an independent predictor of survival in multivariable Cox regression models, although in the better-fit random survival forest model (C-index = 0.735), an ACCI ≥ 6 had a modest contribution to overall survival (mean decrease in the C-index = 0.026), suggesting that its relevance may be more detectable in nonlinear, machine learning-based survival models. This discrepancy also highlights potential methodological and biological considerations.

Similarly, Barz et al. (2022) [22] reported that the ACCI was not a significant prognostic factor in a cohort of 123 glioblastoma patients who underwent surgery for first recurrence. Instead, their study confirmed that the preoperative KPSS and extent of resection were the only independent predictors of survival, further reinforcing the notion that functional status and surgical factors play a greater role in survival than does the comorbidity burden alone.

Our observed trends are in line with current clinical evidence and guideline recommendations. As shown in the CCTG CE.6/EORTC 26,062 − 22,061 trial [23], elderly HGG patients may benefit from the addition of temozolomide to short-term RT, particularly when the tumor exhibits MGMT promoter methylation. According to the EANO guidelines [3], treatment decisions for older or frail patients should prioritize molecular markers and functional status over chronological age. When we extended the multivariate model for hRT and normoRT, MGMT promoter methylation was significantly associated with both improved OS and PFS. In these models, the ACCI approached borderline significance but remained nonsignificant overall. When stratifying by the ACCI, survival differences between CRT strategies varied. For example, hRT alone was associated with significantly worse OS in patients with an ACCI ≤ 5, whereas trends toward benefit from concurrent hRT with TMZ were observed in patients with an ACCI ≥ 6. However, the ACCI alone was not an independent predictor of OS in these treatment comparisons. The KPS was not identified as a significant predictor of OS or PFS, which may reflect current practices where treatment decisions rely on multiple factors - including the MGMT status - rather than the KPS alone. Moreover, accurately assessing the KPS can be particularly challenging in patients with neurological diseases and deficits, which may limit its reliability in this context. Importantly, in this setting, treatment allocation in our cohort reflected real-world clinical practice: hypofractionation was more frequently selected for older or frail patients with higher comorbidity burden. This introduces potential selection bias; however, in our stratified analyses ACCI did not significantly correlate with survival differences after adjusting for RT type, indicating that comorbidity burden itself rather than treatment allocation was the main driver of outcomes.

Thus, the ACCI was not consistently associated with survival across our models. It may play a role in real-world treatment allocation, but our results do not support its use as an independent prognostic marker. These findings support its role more as a triage tool than as a robust prognostic marker once therapy has begun, which aligns with EANO’s position on individualized treatment pathways. Wick et al. (2017), for example, propose stratification via multiple functional assessments, including the KPS, geriatric assessment, and frailty scales. As many trials exclude elderly patients with comorbidities, emphasis should therefore be placed on real-world studies that guide nuanced treatment planning [24].

In addition to comorbidity and treatment allocation, we also examined post-RT impairments. Motor disorders occurred in nearly one in five patients, and epilepsy and thrombocytopenia affected close to 10%, highlighting a substantial clinical burden even in the absence of significant associations with OS or PFS. These impairments can profoundly affect functional independence and quality of life, underscoring the importance of supportive care and monitoring. This aligns with reports from re-irradiation studies in recurrent HGG, where nearly half of patients experiencing disease control developed late toxicities such as radionecrosis and irreversible white matter changes, which—while not always survival-limiting—represent considerable long-term morbidity [25]. Notably, toxicity rates were broadly comparable across treatment strategies in our cohort, suggesting that neither hypofractionation nor addition of temozolomide disproportionately increased adverse events.

To avoid redundancy, we explicitly separated models into ACCI-only and component-only approaches. This addresses the apparent inconsistency of excluding age while including diabetes: diabetes was modeled only in analyses where ACCI was excluded, reflecting its high prevalence in our cohort and its strong association with survival. Sensitivity checks (CCI without age + age; ACCI without diabetes + diabetes) supported the robustness of this approach, showing that the adverse prognostic effect was attributable to diabetes itself rather than the composite score.

While the ACCI showed limited prognostic value for OS and was not significantly associated with PFS, this suggests that it primarily affects long-term survival rather than early disease progression. Diabetes, however, consistently predicted both OS and PFS, underscoring the importance of analyzing specific comorbidities rather than relying solely on composite indices.

The ACCI may also reflect broader health status and treatment tolerability, which are not fully captured in Cox models. In contrast, the RSF model, which handles collinearity and nonlinearity more effectively, identified the ACCI as modestly relevant but less influential than age, MGMT status, or recurrence treatment.

A recent genetic study suggested a potential causal relationship between diabetes incidence and GBM incidence [26]. However, the impact of diabetes on GBM survival remains complex and may depend on factors such as glycemic control, treatment interactions, and underlying metabolic pathways. Our findings are in line with those of previous research suggesting that diabetes may be associated with worse survival according to univariate analyses but was not significant according to adjusted models [27]. This prior review highlights glycemic control rather than diabetes itself as a potential driver of survival outcomes in GBM patients, which we could not assess in our study because of a lack of blood glucose data. This raises the possibility that the prognostic effect of diabetes observed in our univariate model may be influenced by variations in glycemic control among patients [28–30].

Additionally, the lack of significance of the ACCI in the Cox model, despite its relevance in machine learning analysis, suggests that the comorbidity burden interacts with other clinical factors in more complex ways than a standard proportional hazards model can capture. This aligns with findings from Barz et al. (2022) [22], who also reported that ACCI was not a significant predictor in multivariate Cox regression, despite its theoretical relevance as a marker of comorbidity burden.

The inclusion of “any tumor” in the ACCI score likely limited variability in our cohort and contributed to its reduced prognostic relevance. This finding is supported by SEER-based studies showing that prior malignancies do not independently impact GBM survival when adjusted for other factors [31]. Refining comorbidity coding - e.g., separating prior malignancy from current cancer - may improve the ACCI’s utility in disease-specific settings.

Our study revealed that the ACCI was a predictor of OS but had a weaker effect on PFS, depending on the tests used. Some studies that investigated various cancer types agree with our findings, where the ACCI was linked to OS but inconsistently linked to PFS [32]. Some suggest that a higher ACCI affects treatment decisions, potentially influencing outcomes [33]. However, as treatment was comparable across our cohort, our results likely reflect the direct impact of comorbidities. This finding suggests that comorbidity burden may influence outcomes, but our analyses do not establish ACCI as an independent or robust predictor in glioblastoma. Tumor-specific factors and molecular markers appear to play a more dominant role. However, some other studies reported an ACCI effect on both OS and PFS [14, 17].

Our study is limited by small subgroup sizes, possible underreporting of comorbidities, and lack of statistical significance of the ACCI in most models. While RSF suggested a modest contribution of comorbidity burden, these findings should be interpreted cautiously and cannot establish ACCI as a clinically useful prognostic tool in this setting. Larger prospective studies are required before the ACCI can be applied in treatment stratification.

## Conclusion

This study suggests that comorbidity burden, as measured by the ACCI, may influence survival in elderly glioblastoma patients, but the ACCI was not consistently significant across our analyses and its prognostic value appears limited compared with molecular and treatment-related factors. Diabetes without complications consistently predicted poorer OS and PFS, highlighting the importance of analyzing specific comorbidities. Future prospective studies with larger cohorts are warranted to validate the role of ACCI and to identify additional biomarkers that may improve prognostic accuracy in this high-risk population.

## Supplementary Information

Supplementary Table 1. Description of ACCI Scores.

Supplementary Table 2. Distribution of comorbidity components (without the age contribution score).

Supplementary Graph 2. Distribution of ACCI scores.

## Data Availability

The datasets analyzed during the current study are not publicly available due to patient privacy and institutional restrictions but are available from the corresponding author on reasonable request.

## Abbreviations

ACCI: Age-adjusted Charlson comorbidity index
CCI: Charlson comorbidity index
CRT: Chemo-radiotherapy
GBM: Glioblastoma
HGG: High-grade glioma
HRT: Hypofractionated radiotherapy
KPS: Karnofsky performance status
NormoRT: Normofractioanted radiotherapy
OS: Overall survival
PFS: Progression-free survival
RT: Radiotherapy
RSF: Random survival Forest
Re-RT: Reirradiation
