# Supplementary material for: Comorbidity burden in elderly high-grade glioma patients: impact on radiotherapy outcomes
Source: BMC Cancer. 2025 Oct 1;25:1496. doi: 10.1186/s12885-025-14957-5 (PMC12490160; doi:10.1186/s12885-025-14957-5)
Supplement: Supplementary file 1 — Supplementary Table 1. Description of ACCI Scores. [file 12885_2025_14957_MOESM1_ESM.docx]

| **Comorbidity** | **Weight** |
| --- | --- |
| Myocardial infarction, congestive heart failure, peripheral vascular disease, cerebrovascular disease, dementia, chronic pulmonary disease, connective tissue disease, ulcer disease, mild liver disease, diabetes without complications | 1 |
| Diabetes with end-organ damage, hemiplegia, moderate to severe renal disease, any tumor, leukemia, | 2 |
| Moderate or severe liver disease | 3 |
| Metastatic solid tumor, AIDS | 6 |
| For each decade over age 40 years:  41-50: +1  51-60: +2  61-70: +3  71-80: +4  >81: +4 | +1 |
