# Supplementary material for: Comorbidity burden in elderly high-grade glioma patients: impact on radiotherapy outcomes
Source: BMC Cancer. 2025 Oct 1;25:1496. doi: 10.1186/s12885-025-14957-5 (PMC12490160; doi:10.1186/s12885-025-14957-5)
Supplement: Supplementary file 2 — Supplementary Table 2. Distribution of comorbidity components (without the age contribution score). [file 12885_2025_14957_MOESM2_ESM.docx]

| **Comorbidities** | **N** |
| --- | --- |
| Myocardial infarction | **2** |
| Congestive heart failure | **1** |
| Peripheral vascular disease | **1** |
| Cerebrovascular disease | **1** |
| Dementia | **1** |
| Chronic pulmonary disease | **0** |
| Connective tissue disease | **2** |
| Ulcer disease | **3** |
| Mild liver disease | **1** |
| Diabetes without complications | **17** |
| Diabetes with end-organ damage | **5** |
| Hemiplegia | **3** |
| Moderate to severe renal disease | **3** |
| Any tumor | **163** |
| Leukemia | **1** |
| Moderate or severe liver disease | **2** |
| Metastatic solid tumor, AIDS | **2** |
