# Supplementary figures and images for: Comorbidity burden in elderly high-grade glioma patients: impact on radiotherapy outcomes
Source: BMC Cancer. 2025 Oct 1;25:1496. doi: 10.1186/s12885-025-14957-5 (PMC12490160; doi:10.1186/s12885-025-14957-5)

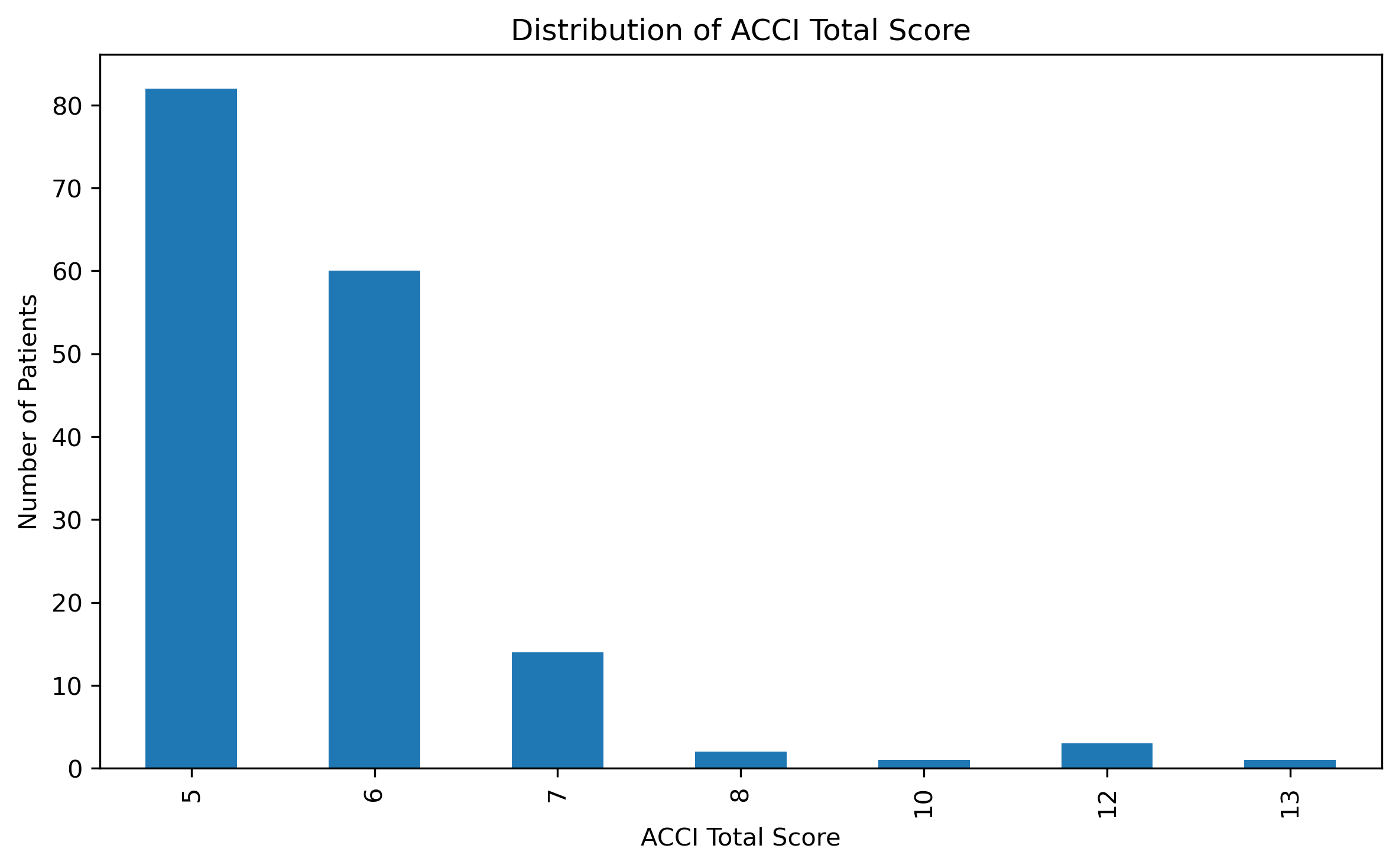

Supplement: Supplementary file 3 — Supplementary Graph 2. Distribution of ACCI scores. [file 12885_2025_14957_MOESM3_ESM.tiff]
